# Vitamin E Supplementation Ameliorates Newcastle Disease Virus-Induced Oxidative Stress and Alleviates Tissue Damage in the Brains of Chickens

**DOI:** 10.3390/v10040173

**Published:** 2018-04-03

**Authors:** Zaib Ur Rehman, Xusheng Qiu, Yingjie Sun, Ying Liao, Lei Tan, Cuiping Song, Shengqing Yu, Zhuang Ding, Muhammad Munir, Venugopal Nair, Chunchun Meng, Chan Ding

**Affiliations:** 1 Shanghai Veterinary Research Institute (SHVRI), Chinese Academy of Agricultural Sciences (CAAS), Shanghai 200241, China; zaib_rehman90@yahoo.com (Z.U.R.); xsqiu@shvri.ac.cn (X.Q.); sunyingjie@shvri.ac.cn (Y.S.); liaoying@shvri.ac.cn (Y.L.); tanlei@shvri.ac.cn (L.T.); scp@shvri.ac.cn (C.S.); yus@shvri.ac.cn (S.Y.); 2 Department of Preventive Veterinary Medicine, College of Veterinary Medicine, Jilin University, Changchun 130062, China; dingzhuang@jlu.edu.cn; 3 Biomedical and Life Sciences, Lancaster University, Lancaster LA1 4YG, UK; muhammad.munir@lancaster.ac.uk; 4 Avian Viral Diseases Programme, The Pirbright Institute, Surrey GU24 0NF, UK; venugopal.nair@pirbright.ac.uk; 5 Jiangsu Co-Innovation Center for Prevention and Control of Important Animal Infectious Diseases and Zoonoses, Yangzhou 225009, China

**Keywords:** Newcastle disease, oxidative stress, histopathology, vitamin E, brain, plasma, glutathione, malondialdehyde, nitric oxide, superoxide dismutase

## Abstract

Newcastle disease (ND), characterized by visceral, respiratory, and neurological pathologies, causes heavy economic loss in the poultry industry around the globe. While significant advances have been made in effective diagnosis and vaccine development, molecular mechanisms of ND virus (NDV)-induced neuropathologies remain elusive. In this study, we report the magnitude of oxidative stress and histopathological changes induced by the virulent NDV (ZJ1 strain) and assess the impact of vitamin E in alleviating these pathologies. Comparative profiling of plasma and brains from mock and NDV-infected chicken demonstrated alterations in several oxidative stress makers such as nitric oxide, glutathione, malondialdehyde, total antioxidant capacity, glutathione S-transferase, superoxide dismutase, and catalases. While decreased levels of glutathione and total antioxidant capacity and increased concentrations of malondialdehyde and nitric oxide were observed in NDV-challenged birds at all time points, these alterations were eminent at latter time points (5 days post infection). Additionally, significant decreases in the activities of glutathione S-transferase, superoxide dismutase, and catalase were observed in the plasma and brains collected from NDV-infected chickens. Intriguingly, we observed that supplementation of vitamin E can significantly reduce the alteration of oxidative stress parameters. Under NDV infection, extensive histopathological alterations were observed in chicken brain including neural inflammation, capillary hyperemia, necrosis, and loss of prominent axons, which were reduced with the treatment of vitamin E. Taken together, our findings highlight that neurotropic NDV induces extensive tissue damage in the brain and alters plasma oxidative stress profiles. These findings also demonstrate that supplementing vitamin E ameliorates these pathologies in chickens and proposes its supplementation for NDV-induced stresses.

## 1. Introduction

A wide range of reactive species are produced as by-products of metabolic processes in the body. These reactive species are broadly classified into two groups of biologically reactive substances: reactive oxygen species (ROS) and reactive nitrogen species (RNS). The ROS include species of high reactivity such as hydroxyl radicals and those of lower reactivity such as superoxide and hydrogen peroxide, whereas the RNS include nitric oxide and peroxynitrite [1]. Controlled production of these reactive species is essential in cell signaling, regulation of cytokines, neuromodulation, transcription, apoptosis, and ion transport [2]. More importantly, reactive species play a vital role in host–pathogen interactions including pathogen recognition, host defense system activation, gene expression, and subsequent adaptation. However, uncontrolled ROS generation causes oxidative tissue damage because these species carry highly reactive and unstable unpaired electrons in their outer electron orbits, which can cause irreversible damage to proteins, lipids, carbohydrates, and nucleic acids [3].

In order to mitigate ROS- and RNS-induced alterations, organisms have acquired well-defined antioxidant defense systems, which comprise enzymatic and nonenzymatic components. Enzymatic components include glutathione peroxidase, catalase, superoxide dismutase, and glutathione reductase, whereas the nonenzymatic system comprises glutathione, thioredoxin, melatonin, carotenoids, vitamin E (VitE), and vitamin C [4]. It has been reported that a number of RNA viruses cause oxidative stresses, including the influenza virus, flaviviridae viruses, the betanoda virus, respiratory syncytial virus, Nipah virus, measles virus, and mesogenic Newcastle disease virus (NDV) [5,6,7,8,9,10,11,12,13]. Dietary supplementation of antioxidants may be a promising approach to diminish economic losses caused by virus-induced oxidative stress.

The NDV belongs to the genus Avulavirus, family *Paramyxoviridae*, and order *Mononegavirales*. With the potential to infect more than 200 species of birds, the virulent strains of NDV cause one of the most infectious diseases of poultry and pose threats to national economies worldwide. Based on the severity of the pathological signs, NDV can be divided into four categories, listed in increasing order of virulence: (1) asymptomatic enteric; (2) lentogenic; (3) mesogenic; and (4) velogenic [14,15]. Based on tissue tropism, velogenic strains of NDV can be further divided into viscerotropic and neurotropic. The viscerotropic strains of NDV mainly cause severe hemorrhagic lesions in the gastrointestinal tract and often lead to neurological signs, whereas neurotropic NDV causes neurological signs along with involvement of the respiratory system [16].

It has been demonstrated that mesogenic and velogenic strains of NDV can replicate in the nervous system [15,17,18,19] causing encephalitis, neuronal necrosis, and neuronal phagocytosis in the grey matter of the brain, cerebellum, and spinal cord [17]. Recent studies conducted by Subbaiah et al. [12,13] have shown that mesogenic NDV can cause oxidative stress in the brain by decreasing the activities of xanthine oxidase, glutathione peroxidase (GPx), glutathione S-transferase (GST), superoxide dismutase (SOD), and catalase (CAT), as well as the levels of glutathione (GSH), uric acid, intracellular protein carbonyls, and nitrates. However, studies regarding the effect of velogenic NDV on oxidative stress and histology in the brains of chickens with marginally protective antibody titer are lacking. ND is mainly controlled by effective vaccination and biosecurity. Historically, the Lasota vaccine strain has been used to protect birds from velogenic NDV-induced mortalities; however, this vaccine fails to provide sterile immunity and birds are required to obtain higher antibody titers to prevent virus shedding [20].

VitE plays an important role in the development of the nervous system [21]. VitE is mainly present in cellular membranes and protects the cells from oxidative stress by scavenging reactive species; it may also be neuroprotective [22]. VitE supplementation has been shown to improve immune function against viral pathogens [22] and can modulate T cell function and cytokines [23]. The NDV causes nervous signs and induces histopathological changes in the brains of chickens; therefore, this study was designed to determine the effect of velogenic NDV on oxidative stress in brains and plasma of chickens and the role of VitE in alleviating these pathologies.

## 2. Materials and Methods

### 2.1. Reagents and Viruses

VitE was purchased from the Aladdine Biotechnology Company (Shanghai, China). The malondialdehyde (MDA), GSH, NO, total antioxidant capacity (TAOC), GST, SOD, CAT, and protein detection kits were procured from Nanjing Jiancheng Biotechnology Research Institute (Nanjing, China).

A wild-type velogenic NDV isolate, ZJ1, was originally isolated from geese in 2000 (Goose/China/ZJ1/2000; GB AF431744.3), and was kindly provided by Professor Xiufan Liu from Yangzhou University (Yangzhou, China). The pathogenicity indices including mean death time, intracerebral pathogenicity index, and intravenous pathogenicity index of the ZJ1 were 51.6, 1.89, and 2.7, respectively [24,25]. The virus stock was prepared by growing the virus in 10-day-old specific-pathogen-free (SPF) embryonated chicken eggs and was subsequently stored at −80 °C until use.

### 2.2. Ethics Statement

This study was carried out in strict accordance with the recommendations in the Guide for the Care and Use of Laboratory Animals of Shanghai Veterinary Research Institute (SHVRI, Shanghai, China), of the Chinese Academy of Agricultural Sciences (CAAS, Beijing, China). All protocols applied in this study were approved by the Institutional Animal Care and Use Committee of SHVRI (Permission number: SHVRI-Ro-2016060092 on 1 June 2016), CAAS, China.

### 2.3. Animal Grouping and Treatments

SPF embryonated chicken eggs were obtained from the Merial Vital Laboratory Animal Technology Company (Beijing, China) and were incubated at the laboratory facility of SHVRI, CAAS. One-day-old SPF chickens were orally vaccinated with LaSota vaccine and were kept in isolators with ad libitum access to feed and water. Sixty-six SPF chickens were selected from the vaccinated chickens based upon antibody titers at seven weeks old and were divided into four groups. Nonsupplemented birds were used as the control group. There were six birds in the control group; all other groups had 20 chickens each. Those birds having hemagglutination inhibition titer of 4–4.25 log_2_ were selected for this experiment. It is well documented that virulent NDV can replicate in birds with lower antibody titers [20,26]. Therefore, we selected birds carrying lower antibody titers to determine the effect of virus replication on the oxidative stress parameters. The birds in Group 1 were the nonsupplemented, nonchallenged control (CON). Chickens in Group 2 (*n* = 20) were supplemented with 200 µL of soybean oil, and abbreviated as NS + NDV. Birds in Groups 3 and 4 (*n* = 20 each) were supplemented with 50 and 100 IU/day/Kg body weight of VitE (α-tocopherol), respectively, dissolved in 200 µL of soybean oil; they were abbreviated as VE50 + NDV and VE100 + NDV, respectively. Administration of VitE was done orally via gavage before the first feeding every day. The chickens in Groups 2, 3, and 4 were challenged by intramuscular route with a ZJ1 suspension containing 10^5.5^ 50% egg lethal dose (EID_50_), seven days after the start of supplementation.

### 2.4. Sample Collection

Four birds per treatment were selected for daily collection of blood and brain samples. Blood samples were collected from the jugular vein at 1, 2, 3, 4, and 5 days post infection (DPI) in heparin tubes and immediately transferred to the laboratory, maintaining the cold chain. Plasma was obtained by centrifugation (2000× *g*) for 10 min at 4 °C. Plasma samples were stored at −80 °C until analysis.

A total of four chickens per experimental treatment were humanly killed by an intravenous injection using pentobarbital sodium (40 mg/kg) every day for the collection of cerebrum brain samples at 1, 2, 3, 4, and 5 DPI. Birds in the control group were killed at 3 and 5 DPI. One part of every brain was put in microtubes (already marked and weighed), immediately frozen in liquid nitrogen, and subsequently stored at −80 °C until use. Another part of each brain was fixed in 10% neutral-buffered formaldehyde tubes for histological studies.

### 2.5. Preparation of Tissue Homogenates

Brain samples were removed from −80 °C storage, weighed, homogenized in nine volumes of ice-cold phosphate-buffered saline (PBS) using a Tissuelyser-24 (Xin Jin Technology, Shanghai, China) at 20 Hz for 45 s, and instantly centrifuged at 3000× *g* at 4 °C for 10 min. The pellet was discarded and the supernatant was preserved for future analysis.

### 2.6. Determination of Protein Content

Protein content was determined following the method of Lowry et al. [27] using bovine serum albumin as a reference standard.

### 2.7. Determination of NO Level

Homogenized brain and plasma samples were used for the determination of NO content, using the NO assay kit (Nanjing Jiancheng Bioengineering Institute, Nanjing, China) and strictly following the manufacturer’s instructions.

### 2.8. Determination of MDA Level

Lipid peroxidation in the plasma and brain homogenates was determined using commercial kits (Nanjing Jiancheng Bioengineering Institute, Nanjing, China), according to the manufacturer’s instructions. Briefly, the mixing of trichloroacetic acid with the homogenate produced a red-colored substance. The reaction mixture was centrifuged, and absorbance of the supernatant was observed at 532 nm using a spectrophotometer (Multiskan™ GO Microplate Spectrophotometer, Thermo Fisher Scientific, Waltham, MA, USA).

### 2.9. Determination of GSH Level

GSH levels in the plasma and brain samples were determined by using a kit (Nanjing Jiancheng Bioengineering Institute, Nanjing, China). The detection procedure was based on yellow color development, produced by the reaction of the sulfhydryl compounds with dithio-dinitrobenzoic acid. Concentration of GSH was determined by measuring the absorbance at 405 nm.

### 2.10. Determination of TAOC

The TAOC activity of the brain and plasma samples was examined using a colorimetry kit (Nanjing Jiancheng Bioengineering Institute, Nanjing, China). The absorbance of the resulting reaction was measured at 520 nm using a spectrophotometer (Multiskan™ GO Microplate Spectrophotometer, Thermo Fisher Scientific).

### 2.11. Determination of CAT Activity

The CAT activity of the plasma and brain samples was spectrophotometrically measured using commercially available kits. CAT decomposes hydrogen peroxide and the reaction can be stopped by adding ammonium molybdate. The remaining H_2_O_2_ reacted with ammonium molybdate and produced a pale-yellow clathrate, which was used to measure the absorbance at 405 nm using a spectrophotometer (Multiskan™ GO Microplate Spectrophotometer, Thermo Fisher Scientific).

### 2.12. Determination of SOD activity

The SOD activity was determined using the nitro blue tetrazolium method [28]. This protocol is based on the ability of the enzyme to inhibit the phenazine-methosulfate-mediated reduction of nitro blue tetrazolium dye. The absorbance of the developed color was determined at 550 nm using a spectrophotometer (Multiskan™ GO Microplate Spectrophotometer, Thermo Fisher Scientific).

### 2.13. Determination of GST Activity

The GST activity was determined by measuring the level of conjugation of GSH with 1-chloro-2,4-dinitrobenzene. The absorbance of the colorable reaction mixture was examined at 412 nm using a spectrophotometer (Multiskan™ GO Microplate Spectrophotometer, Thermo Fisher Scientific).

### 2.14. NDV Antibody Titers

For the determination of NDV antibody titers, an enzyme-linked immunosorbent assay kit was purchased from Yu Song Biotech Company (Shenzhen, China). Plasma samples were diluted 40 times and incubated in a 96-well plate coated with the NDV antigen. The assay was performed strictly according to manufacturer’s instructions.

### 2.15. Detection of Viral Loads in the Brain

The total RNA was extracted from the brain samples using TRIzol (Invitrogen, Carlsbad, CA, USA) following the manufacturer instructions. The quality of the RNA was examined by using NanoDrop™ 2000/2000c (Thermo Fisher Scientific Inc., Wilmington, DE, USA) for the ratio of 260 nm versus 280 nm. Next, the RNA was reverse transcribed to cDNA using M-MLV reverse transcriptase (Promega, Madison, WI, USA) with the 6-nt random primer according to the manufacturer’s instructions. Real-time quantitative polymerase chain reaction (qRT-PCR) was performed to determine the expression of the ZJ1 M gene, and the β-actin housekeeping gene was used for normalization using SYBR Premix Ex Taq reagents (Takara, Dalian, China) in the CFX96 real-time PCR detection (CFX96; Bio-Rad, Hercules, CA, USA) system. The primers used for the viral M gene were Forward: 5′-TACTTTGATTCTGCCCTCCCTT-3′ and Reverse: 5′-TAAGCAGAGCATTGCGGAAGA-3′, and those for the β-actin gene were: Forward: 5′-ATTGTCCACCGCAAATGCTTC-3′ and Reverse: 5′-AAATAAAGCCATGCCAATCTCGTC-3′. The expression of the target gene was normalized with the β-actin after ensuring the same amplification efficiency.

### 2.16. Histopathology

The samples of chicken cerebrum were collected and immediately fixed in a neutral-buffered formalin solution (10% *v*/*v*). These fixed tissue samples were dehydrated through a graded series of ethanol, cleaned by xylene, embedded in paraffin wax, and sectioned. Finally, the sections were stained with hematoxylin and eosin and observed using light microscopy.

### 2.17. Statistical Analysis

Statistical analysis was performed using one-way analysis of variance with SPSS software (Version 17.0, SPSS Inc., Chicago, IL, USA). All measurements were replicated four times. The Duncan multiple range test was used to determine the significant differences between different treatments. The differences were considered significant if *p* < 0.05.

## 3. Results

### 3.1. Antioxidants and Associated Enzymes

Several parameters such as nonenzymatic antioxidants (NO, GSH, MDA, and TAOC) and enzymatic antioxidants (CAT, SOD, and GST) were determined in the brain homogenates and plasma. The effects on a variety of parameters were monitored in the brain.

#### 3.1.1. Nonenzymatic Antioxidant Parameters

**Figure 1:**
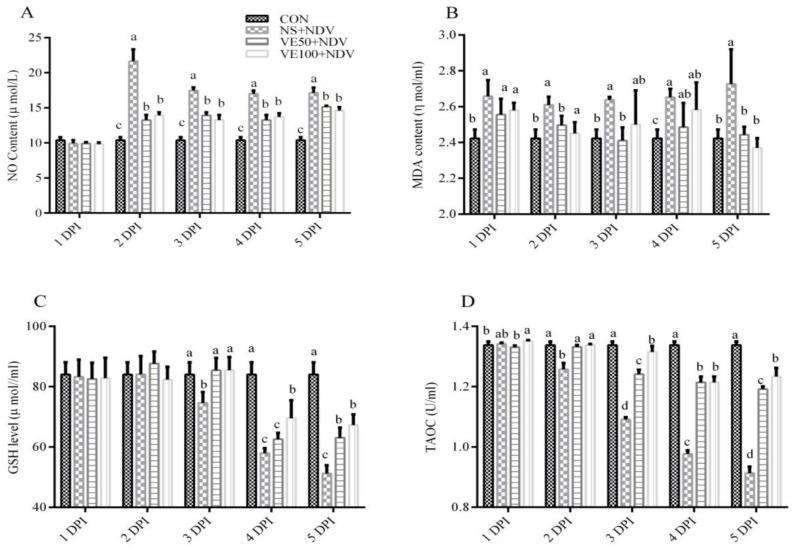
Effects of Newcastle disease virus (NDV) and vitamin E (VitE) supplementation on nonenzymatic antioxidants in plasma of chickens. Plasma samples were prepared from uninfected and NDV-infected chickens, which were treated or untreated with different doses of vitamin E to determine (**A**) NO levels; (**B**) malondialdehyde (MDA) levels; (**C**) GSH levels; and (**D**) total antioxidant capacity (TAOC). Significant differences were observed between different treatments at different time points monitored at 1, 2, 3, 4, and 5 DPI. Data is expressed as mean ± standard error of mean. Columns marked with different superscripts (a–d) are significantly different (*p* < 0.05).

**Figure 2:**
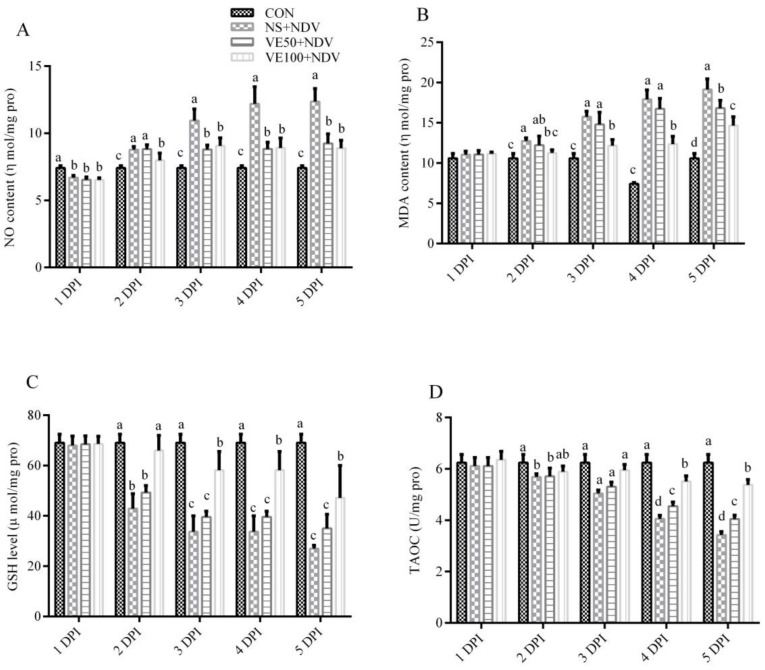
Effects of NDV and VitE supplementation on nonenzymatic antioxidants in brains of chickens. Brain homogenates were prepared from the samples collected at 1, 2, 3, 4, and 5 DPI from different treatments to determine (**A**) NO levels; (**B**) MDA levels; (**C**) GSH levels; and (**D**) TAOC. Significant differences were observed between different treatments at different time points monitored at 1, 2, 3, 4, and 5 DPI. Data is expressed as mean ± standard error of mean. Columns marked with different superscripts (a–d) are significantly different (*p* < 0.05).

The effect of VitE supplementation on the nonenzymatic antioxidant parameters in the brain and plasma is shown in Figure 1 and Figure 2. The NO contents were markedly higher in NDV-challenged birds at 2, 3, 4, and 5 DPI compared with in the control (*p* < 0.05). This increase in NO content of the brain and plasma was less in VitE-supplemented groups compared with in the NS + NDV group.

The MDA contents in the brain and plasma were significantly (*p* < 0.05) higher in NDV-challenged groups compared with in nonchallenged controls. The increase in MDA content in the plasma and brain was offset in groups VE50 + NDV and VE100 + NDV; VitE supplementation at higher doses was most effective.

The GSH contents and the TAOC in the brain and plasma were determined in NDV-infected and VitE-supplemented birds (Figure 1C,D and Figure 2C,D). Marked decreases in GSH content and TAOC were observed in the NS + NDV group compared with in the CON group. This reduction was more severe at 5 DPI compared with at 2 DPI. In the VE50 + NDV and VE100 + NDV groups, the decreases in GSH and TAOC were reversed due to supplemental VitE.

#### 3.1.2. Enzymatic Antioxidant Parameters

**Figure 3:**
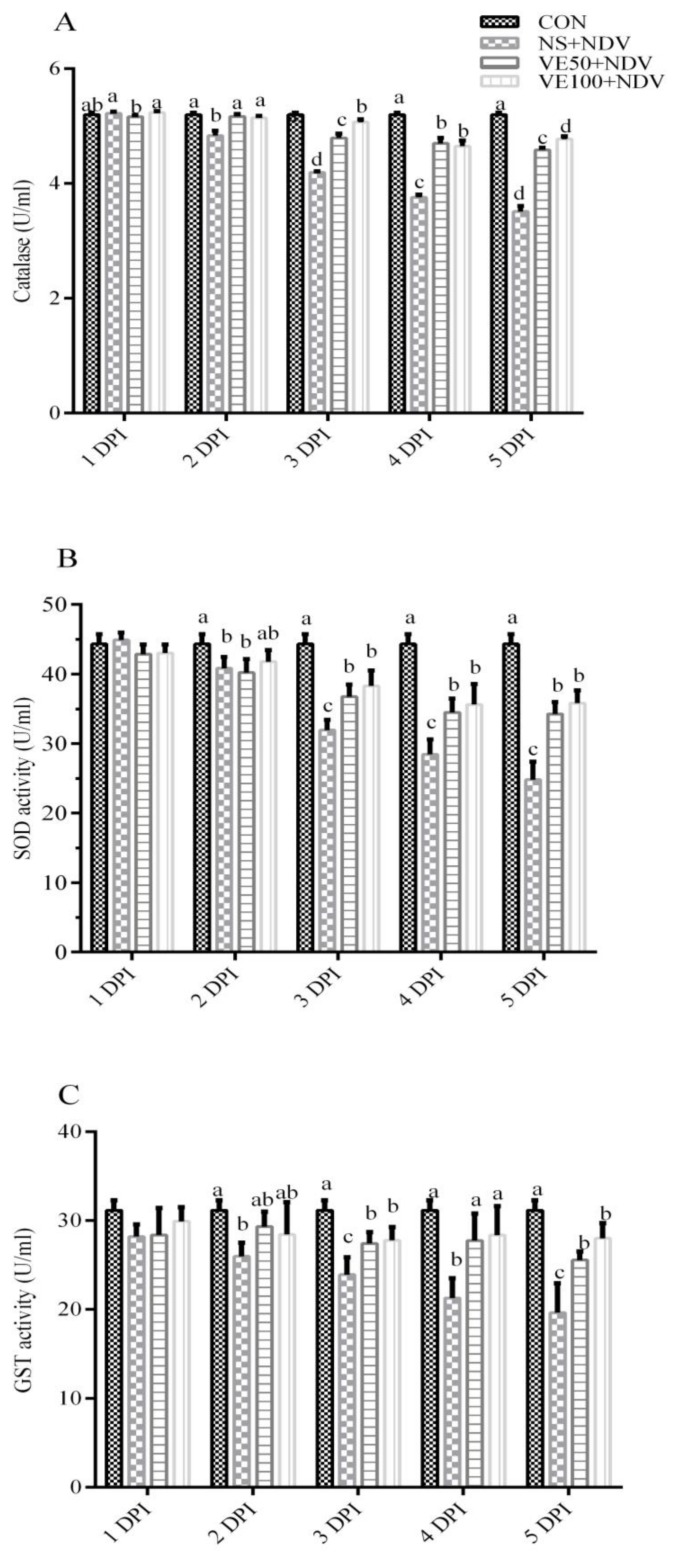
NDV infection inhibits the activity of antioxidant enzymes in plasma of chickens. Specific biological assays were performed to determine the activity of (**A**) catalase (CAT); (**B**) superoxide dismutase (SOD); and (**C**) glutathione S-transferase (GST) in NDV-infected chicken plasma. Blood was collected from NDV-infected and uninfected chickens, supplemented or nonsupplemented with different doses of VitE at 1, 2, 3, 4, and 5 DPI. Data is expressed as mean ± standard error of mean. Columns marked with different superscripts (a–d) are significantly different (*p* < 0.05).

**Figure 4:**
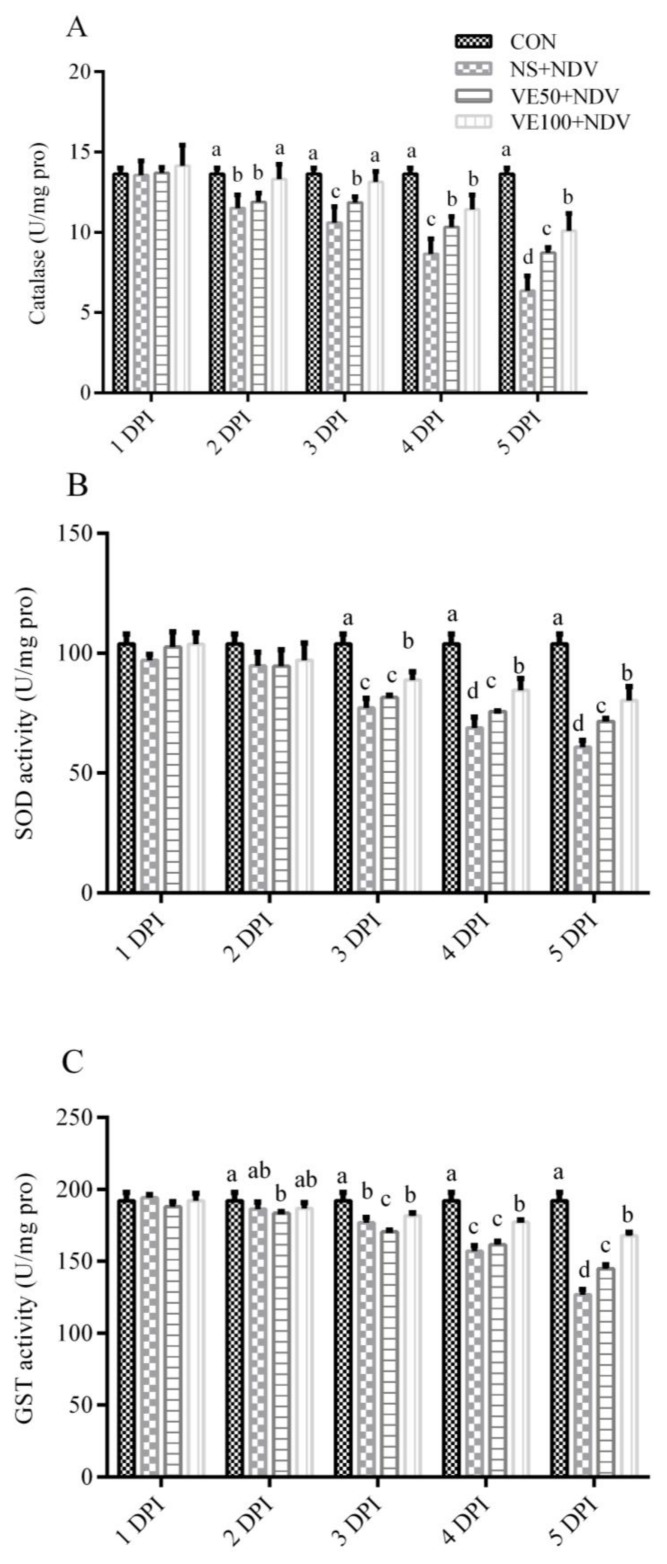
NDV infection inhibits the activity of antioxidant enzymes in the brain. The activity of (**A**) SOD; (**B**) CAT; and (**C**) GST in NDV-challenged and nonchallenged chicken brains. Brain homogenates were prepared from NDV-challenged and nonchallenged chickens, supplemented or nonsupplemented with different doses of VitE at 1, 2, 3, 4, and 5 DPI. Data is expressed as mean ± standard error of mean. Columns marked with different superscripts (a–d) are significantly different (*p* < 0.05).

The catalase activities were significantly lower (*p* < 0.05) in NDV-challenged groups than in the control group (Figure 3A and Figure 4A). CAT activity decreased with increases in DPI; in other words, it was more pronounced at 5 DPI compared with at 2 DPI. CAT activities were less decreased in the VE50 + NDV and VE100 + NDV groups.

As shown in Figure 3 and Figure 4B,C, NDV-infected groups had significant decreases in SOD and GST activities in the brain and plasma samples compared with the control birds. Interestingly, lower decreases in SOD activities were found in the VE50 + NDV and VE100 + NDV groups at 3, 4, and 5 DPI.

### 3.2. Virus Load and Antibody Titers

**Figure 5:**
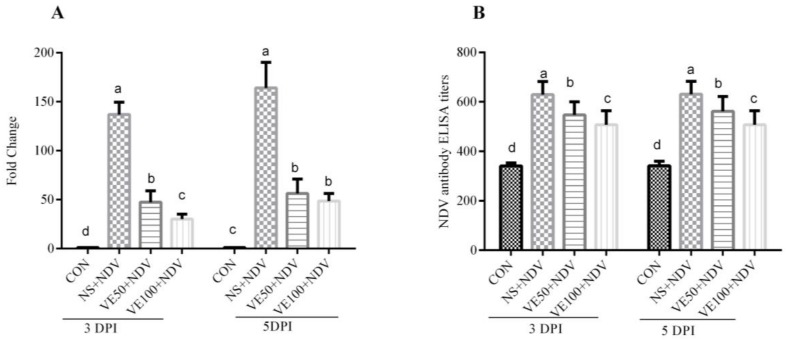
Determination of NDV loads and ELISA titers. (**A**) NDV loads. Samples were collected at 3 and 5 DPI, and the M gene was targeted to quantify NDV replication in the intestine of tested chickens; (**B**) NDV antibody titers. Plasma samples from chickens were collected at 3 and 5 DPI and NDV antibody titers were determined. Bars with a common superscript letter (a–d) are not significantly different (*p* ≤ 0.05).

To determine the viral load in the brain samples, we examined the transcriptional level of the virus M gene using quantitative RT-PCR. NDV load was detected at 3 and 5 DPI (Figure 5A). Virus load in the brain of the NS + NDV group was significantly higher (*p* < 0.05) than those of the VE50 + NDV and VE100 + NDV groups.

Blood was collected at 3 and 5 DPI to determine the antibody titers. Birds in the control group had antibody titers ranging from 320 to 370. Increases in the antibody titers of all the NDV challenge groups were observed. These increases in antibody titers were significantly (Figure 5B) different between the VitE supplemented and nonsupplemented, NDV-challenged groups.

### 3.3. Brain Histopathology

**Figure 6:**
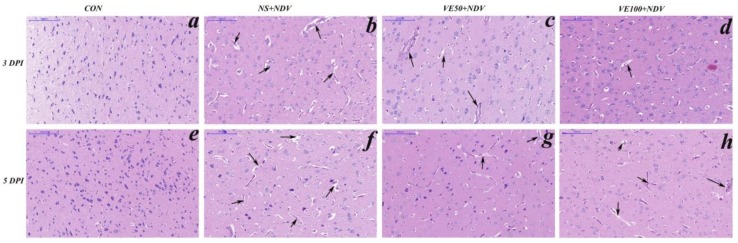
Histopathological changes in the cerebrum part of the brain tissue of chicken (hematoxylin and eosin staining). Panels (**a**–**d**) show the histopathology of CON, NS + NDV, VE50 + NDV, and VE100 + NDV groups, respectively, at 3 DPI. Panels (**e**–**h**) demonstrate the histopathological changes of CON, NS + NDV, VE50 + NDV, and VE100 + NDV groups, respectively, at 5 DPI. No histopathological change was observed in the control birds (**a**,**e**). Inflamed and damaged neurons and capillary hyperemia can be seen in the NDV-infected groups, but these histopathological changes were less pronounced in vitamin-E-supplemented groups (**c**,**d**,**g**,**h**). Histopathological changes were more severe at 5 DPI compared with at 3 DPI.

The histopathological study of the cerebrum samples showed morphological changes induced by the NDV challenge (Figure 6). The control group showed normal morphology (Figure 6a,e), but some degree of encephalitis was present in all NDV-challenged groups. Severe histological alterations, including clusters of cells with microglial morphology, neural inflammation, capillary hyperemia, necrosis, and loss of prominent axons were observed in NS + NDV birds; inevitably, it was more severe at 5 DPI (Figure 6b,f). When compared with the control, histological changes were less pronounced in the VitE-supplemented NDV-challenged groups; the majority of neurons were normal in the VE100 + NDV group compared with in VE50 + NDV. In general, NDV-induced morphological changes in the neurons and VitE supplementation attenuated these effects.

## 4. Discussion

It is well known that NDV can replicate efficiently in the brains of chickens [15,17]; however, the replication ability varies among different NDV isolates. The peripheral rate of virus replication and the ability to spread into nervous tissue affects the nervous signs and microscopic lesions in the brain [15].

The brain is a soft organ and is extremely susceptible to oxidative stress induced by ROS and RNS. The brain contains a large quantity of poly-unsaturated fatty acids and utilizes 20% of the body’s oxygen. It produces a large amount of ROS due to high concentrations of those compounds that are catalytically involved in the production of free radicals [29], and a comparatively poor enzymatic antioxidant defense system [30]. Other factors which may contribute to the increased production of ROS in the brain are due to high aerobic metabolism and blood perfusion [31].

In this study, we determined the effect of virulent NDV on oxidative stress makers, virus load, antibody titer, and histopathology in the brains and plasma of chickens with or without supplementation of VitE. Recently, Subbaiah et al. [12,13] showed that infection of chickens with mesogenic NDV induces ROS and RNS production. However, studies explaining the daily picture of ROS and RNS production in the brains and plasma of velogenic NDV-infected chickens are not available. Our findings of the progressive increase in lipid peroxidation, as determined by the MDA, were observed along with increased production of NO.

NO is an important metabolite and plays vital roles in the host defense system by recruiting immune cells across vascular epithelial barriers during mild to moderate infections [32]. However, higher production of NO causes decreased protein function, along with tissue and DNA damage. Overproduction of NO increases pathogenicity, while its inhibition increases the survival rate in influenza and human paramyxovirus respiratory syncytial virus infections [33,34,35]. We observed higher levels of NO in the brains and plasma of chickens infected with virulent NDV. This increase was less in VitE-supplemented birds. Our results are in accordance with studies demonstrating that NDV can induce NO in peripheral blood mononuclear cells [36], hetrophils [37], spleen, and sera of chickens [32]. Higher production or levels of NO may be a contributing factor in the increased mortality in ND because it increases virus replication [38].

Many viral diseases cause neurodegeneration by disturbing the oxidative balance. The most common viruses that adversely affect the oxidative defense system in the brain are Japanese encephalitis virus [39], reptarena virus [40], human immunodeficiency virus type-1 [4], and herpes simplex virus [41]. Lipid peroxidation is one of the most important factors in oxidative stress in which the free radical production occurs mainly in the lipid membranes. The brain is rich in poly-unsaturated fatty acids, the main target of lipid peroxidation, resulting in the production of highly reactive aldehydes [42]. These reactive aldehydes, including MDA, cause damage to cellular homeostasis and interfere with proteins by the process of Michael adduction [42]. GSH is the major cellular nonenzymatic antioxidant and plays a pivotal role in detoxifying different metabolites and maintaining the redox balance [43]. SOD, GST, and CAT are the primary enzymes of the antioxidant defense system, responsible for free radical elimination and oxidative stress prevention. In this experiment, we found an increase of MDA and decreases of GSH and TAOC contents in the brain and plasma of NDV-challenged birds. This increase was more obvious in NS + NDV birds compared with in VitE-supplemented groups. These results are in agreement with Subbaiah et al. [12,13], which demonstrated that mesogenic NDV infection induces oxidative stress in the brain and liver of chickens. Our results indicated that virulent NDV infection in chickens decreases the activities of vital enzymes, including SOD, GST, and CAT, involved in the elimination of ROS and the maintenance of redox balance. Numerous previous studies showed that other paramyxoviruses, like respiratory syncytial virus [6], Nipah virus [11,44], measles virus [10], and Sendai virus [45], cause oxidative stress.

Histopathological observations are the best way to determine the degree of tissue damage. It is reported that NDV causes morphological changes in different parts of the brain in poultry [15,17,18,19]. In this study, NDV infections markedly disorganized the morphological structure of the cerebrum, manifesting neural inflammation, capillary hyperemia, axonal degeneration, and necrosis. Our results showed that increased levels of NO and MDA and decreased levels of cellular GSH and TAOC, along with decreased activity of SOD, GST, and CAT, were the most probable bases of histopathological changes, and were associated with high virus replication. Decreased concentrations of antioxidants like GSH have been reported to be involved in many brain disorders [43]. In this study, less pathological alteration was found in the brains of VitE-supplemented birds. This might be due to its properties, like the presence of VitE in the cellular membranes, chain-breaking antioxidants, and the inhibition of lipid peroxidation in membranes [46].

## 5. Conclusions

In conclusion, our data indicates that virulent NDV infection in chickens causes oxidative stress in the brain and plasma by lowering the levels of GSH and TAOC, decreasing the activities of CAT, SOD, GST, and increasing the levels of MDA and NO. Additionally, VitE supplementation alleviates oxidative stress. This study also demonstrated that oxidative stress contributes to histopathological disorders of brains and was associated with higher virus loads. Further studies are needed to advance our understanding of the basic mechanisms involved in oxidative damage induced by the NDV infection.
